# Trimetazidine Attenuates Cardiac Dysfunction in Endotoxemia and Sepsis by Promoting Neutrophil Migration

**DOI:** 10.3389/fimmu.2018.02015

**Published:** 2018-09-04

**Authors:** Jing Chen, Bei Wang, Jinsheng Lai, Zachary Braunstein, Mengying He, Guoran Ruan, Zhongwei Yin, Jin Wang, Katherine Cianflone, Qin Ning, Chen Chen, Dao Wen Wang

**Affiliations:** ^1^Division of Cardiology, Department of Internal Medicine, Tongji Hospital, Tongji Medical College, Huazhong University of Science and Technology, Wuhan, China; ^2^Hubei Key Laboratory of Genetics and Molecular Mechanisms of Cardiological Disorders, Wuhan, China; ^3^Department of Rheumatology and Immunology, Tongji Hospital, Tongji Medical College, Huazhong University of Science and Technology, Wuhan, China; ^4^Department of Internal Medicine, The Ohio State University, Columbus, OH, United States; ^5^Centre de Recherche de l'Institut Universitaire de Cardiologie & Pneumologie de Québec, Université Laval, Québec, QC, Canada; ^6^Department of Infectious Disease, Institute of Infectious Disease, Tongji Hospital, Tongji Medical College, Huazhong University of Science and Technology, Wuhan, China

**Keywords:** pyroptosis, trimetazidine, septic cardiac dysfunction, neutrophil, AMPK-Nrf2-CXCR2 axis

## Abstract

**Aims:** Cardiac dysfunction can be a fatal complication during severe sepsis. The migration of neutrophils is significantly impaired during severe sepsis. We sought to determine the role of trimetazidine (TMZ) in regulation of neutrophil migration to the heart in a mouse model of sepsis and endotoxemia, and to identify the mechanism whereby TMZ confers a survival advantage.

**Methods and Results:** C57/BL6 mice were (1) injected with LPS followed by 24-h TMZ administration, or (2) treated with TMZ (20 mg/kg/day) for 1 week post cecal ligation and puncture (CLP) operation. Echocardiography and Millar system detection showed that TMZ alleviated cardiac dysfunction and histological staining showed the failure of neutrophils migration to heart in both LPS- and CLP-induced mice. Bone marrow transplantation revealed that TMZ-pretreated bone marrow cells improved LPS- and CLP-induced myocardial dysfunction and enhanced neutrophil recruitment in heart. In CXCL2-mediated chemotaxis assays, TMZ increased neutrophils migration via AMPK/Nrf2-dependent up-regulation of CXCR2 and inhibition of GRK2. Furthermore, using luciferase reporter gene and chromatin immunoprecipitation assays, we found that TMZ promoted the binding of the Nrf2 and CXCR2 promoter regions directly. Application of CXCR2 inhibitor completely reversed the protective effects of TMZ *in vivo*. Co-culture of neutrophils and cardiomyocytes further validated that TMZ decreased LPS-induced cardiomyocyte pyroptosis by targeting neutrophils.

**Conclusion:** Our findings suggested TMZ as a potential therapeutic agent for septic or endotoxemia associated cardiac dysfunction in mice.

**STUDY HIGHLIGHTS**

What is the current knowledge on the topic?

Migration of neutrophils is significantly impaired during severe sepsis, but the underlying mechanisms remain unknown.

What question did this study address?

The effects of TMZ on cardiac dysfunction via neutrophils migration.

What this study adds to our knowledge

TMZ attenuated LPS-induced cardiomyocyte pyroptosis and cardiac dysfunction by promoting neutrophils recruitment to the heart tissues via CXCR2.

How this might change clinical pharmacology or translational science

Our findings suggested TMZ as a potential therapeutic agent for septic cardiac dysfunction.

## Key points

1. TMZ attenuates cardiac dysfunction via neutrophils migration
2. TMZ decreases cardiomyocyte pyroptosis by targeting neutrophils

## Introduction

Sepsis is a life-threatening organ dysfunction caused by a dysregulated host response to infection, and is one of the most common causes of death in hospitalized patients (1). So far, the therapeutic options for sepsis are nonspecific and are limited to support of organ function. In addition, there are still no approved drugs that specifically target sepsis (2). One of the major complications of sepsis, myocardial dysfunction, contributes significantly to increased mortality (3). However, the precise mechanisms that cause myocardial dysfunction during sepsis remain incompletely understood (3). Thus, elucidation of the pathophysiologic processes of sepsis-induced myocardial dysfunction, and seeking new specific drugs, may develop more effective therapies to treat it.

Neutrophil migration into infection sites constitutes the first line of defense against infection (4). The failure of neutrophil migration to the infection site is associated with increased severity of illness and multi-organ dysfunction during septic shock (5, 6). Neutrophil recruitment to the infection site is dependent on the CXC chemokines (7). In murine, CXC chemokines regulate the recruitment of neutrophils via a specific seven-transmembrane type G protein-coupled receptor, CXCR2, while in humans it is dependent on both CXCR1 and CXCR2 (8). However, in some pathological conditions, phosphorylation of CXCR2 by the G protein-coupled receptor kinase 2 (GRK2) triggered receptor desensitization and internalization, resulting in reduced expression of CXCR2 on the surface of neutrophils (9). Previous studies have revealed that the decreased expression of CXCR2 impaired neutrophil recruitment to the infection site and played a major role in the poor outcome secondary to the sepsis (10). Hence, it is necessary to investigate the regulation of neutrophil recruitment during sepsis-induced myocardial dysfunction.

AMPK, 5′ adenosine monophosphate-activated protein kinase, is a crucial regulator of cellular energy homeostasis (11). A recent study has reported that activated AMPK enhanced the abilities of neutrophil chemotaxis and bacterial killing in sepsis (12). Nuclear factor erythroid-2-related factor-2 (Nrf2), which is one of the downstream signals of AMPK, is a critical transcriptional factor for antioxidation. We, and others, have found that activation of Nrf2, via AMPK, inhibited LPS-induced inflammatory response (13, 14). Upon activation, Nrf2 dissociates from Kelch-like ECH-associated protein 1 (Keap1), and in turn, translocates into the nucleus to bind to the antioxidant responsive element (ARE) in gene promoters (15). Nrf2 was found to regulate the expression of many antioxidant enzymes and proteins, such as NADPH quinineoxidoreductase-1 (NQO-1), heme oxygenase-1 (HO-1), and glutathione S-transferase (GST). Recently, it has been indicated that Nrf2 also transcriptionally regulates inflammation-related genes (16).

Trimetazidine (TMZ) is a clinically effective anti-anginal agent due to the inhibition of long-chain 3 ketoacyl coenzyme A thiolase activity, which leads to decreased fatty acid oxidation and increased glucose oxidation (17). Previous studies have indicated the protective effects of TMZ on heart failure (18), oxidative stress damage (19), cell apoptosis (14), and endothelial function (20). Our recent study has demonstrated that TMZ improves LPS-induced cardiac dysfunction by regulating the function of macrophages (14). Given the pivotal function of neutrophils in inflammatory response, the detailed role of TMZ in regulating neutrophils function during septic cardiac dysfunction need to be explored.

Pyroptosis is a form of inflammatory programmed cell death (PCD). Unlike apoptosis or necrosis, pyroptosis features pore formation of the plasma membrane, cell swelling, and membrane rupture, causing leakage of cytosolic contents (21). During LPS-induced pyroptosis, caspase-11 is first activated by directly binding to LPS (22). Subsequently, activated caspase-11 processes interleukin (IL)-1β/IL-18 into their active forms (23). Pyroptosis was initially defined as an antimicrobial reaction in immune cells (24). However, few studies have focused on cardiomyocyte pyroptosis in septic cardiac dysfunction.

In this study, we demonstrated that TMZ ameliorated LPS- and cecal ligation and puncture (CLP)-induced cardiac dysfunction and cardiomyocyte pyroptosis by promoting CXCR2-dependent neutrophil migration to cardiac tissue.

## Materials and methods

### Reagents

LPS, SB225002, Percoll, and Compound C (CC) were from Sigma-Aldrich (St. Louis, MO). TMZ was from Servier (Tianjin, China). RPMI1640 medium was from Thermo Fisher Scientific (Walham, MA). Nrf2 siRNA was from RiboBio (Guangzhou, China). CXCL2 was from R&D Systems (Minneapolis, MN). DAPI and IL-1β ELISA kit were from Boster (Wuhan, China). Myeloperoxidase (MPO) assay kit and lacate dehydrogenase (LDH) assay kit were from Nanjing Jiancheng Bioengineering Institute (Nanjing, China). Lipofectamine 2000 was from Invitrogen (Waltham, MA). Protein A/G agroase, anti-Nrf2, and anti-CXCR2 were from Santa Cruz Biotechnology (Dallas, TX). Anti-Ly6G, anti-MPO, anti-GRK2, and isotype antibody were from Abcam (Cambridge, MA). Anti-AMPK and anti-phospho-AMPK were from Cell Signaling Technology (Danvers, MA). Anti-caspase-11 was from Novus Biologicals (Littleton, CO). FITC-CD11b and Percp/Cy5.5-Ly6G were from eBioscience (San Diego, CA). PE-CXCR2 was from BD Biosciences (San Jose, CA). Lysis buffer and BCA protein assay were from Beyotime (Shanghai, China).

### Animals

All experiments were performed with the approval of the Animal Research Committee of Tongji Medical College, and in accordance with ARRIVE and NIH guidelines for animal welfare. Male C57BL/6 mice at the age of 8–10 week-olds were purchased from the Institutional Animal Research Committee of Tongji Medical College, housed at a temperature of 23–25°C and a humidity of 55 ± 5% with free access to food and water. (1) Model of LPS-induced endotoxemia: Mice were randomly divided into 4 groups (*n* = 8), and received saline, single TMZ, single LPS, or LPS and TMZ combination interventions, respectively. In LPS and TMZ combination interventions, mice were first injected with LPS (15 mg/kg) in 100 μl of sterile saline by intraperitoneal injection, then 6 h later, TMZ (20 mg/kg) was administrated intragastrically (i.g.) every 6 h for a total of three times. In the 6-group animal experiments, the CXCR2 antagonist SB225002 (10 mg/kg) was injected intraperitoneally 30 min before LPS injection (10). (2) Model of CLP sepsis: The mice (*n* = 10 in each group) were first pre-treated with TMZ for 1 day. Then the CLP model of sepsis of moderate severity was performed in accordance with the original protocol developed by Chaudry's lab, with additional modifications (25). In brief, mice were anesthetized with i.p. injection of sodium pentobarbital (Sigma-Aldrich) with a dose of 30 mg/kg. A midline incision was made, and after externalization, the cecum was ligated (1 cm from the apex) and punctured twice (through-through) with a 27-G needle. Next, a small amount of fecal mass from the punctured cecum was gently squeezed out to ensure patency of punctures, cecum was relocated, and 4/0 sutures were used to close the peritoneum and skin. Sham-operated mice underwent only incision and cecum exteriorization. After the sham or CLP operations, the mice were then treated with TMZ (20 mg/kg/day) for 6 consecutive days. The survival rate was determined daily for 7 days after CLP. Cardiac function of mice was assessed by echocardiography and Millar catheter, and then the mice were sacrificed. Part of heart tissue was kept in 10% formalin, dissected and cut into slices. The remaining portions of heart tissue was immediately snap-frozen and stored at −80°C for western blotting examination.

### Bone marrow transplantation

We performed bone marrow transplantation in TMZ or vehicle treated-wild type (WT) C57BL/6 mice using previously established methods (26). Briefly, 10-week-old WT C57BL/6 donor mice were pre-treated using TMZ (20 mg/kg in saline solution for 3 days) or equal amount of solvent (saline solution) as vehicle (TMZ BM and Vehicle BM groups in donor mice), meanwhile, the recipient mice were also pre-treated using TMZ (20 mg/kg in saline solution for 3 days) or equal amount of solvent (saline solution) as vehicle (TMZ and Vehicle groups in recipient mice). Then before the BM transplantation, the recipient mice received 850 rads of γ-irradiation and were administered with the antibiotic, Baytril. The next day, fresh bone marrow cells were isolated from a separate cohort of saline pre-treated C57BL/6 vehicle mice and nonirradiated TMZ pre-treated mice (n = 5 mice/group and pooled), respectively, and were injected into irradiated mice (6 × 10^6^) in 200 μL volume through the tail vein. Twenty-four hours after bone marrow transplantation, the mice were subjected to LPS injection (15 mg/kg) or CLP surgery. Six hours after LPS administration and 1 day after CLP, mice hearts were harvested for immunohistochemistry Ly6G, MPO staining, and other tests.

### Echocardiography and haemodynamic analyses

Transthoracic echocardiography (Vevo3100, Visual Sonics, Canada) was performed under anesthesia (2% isoflurane) (27). For the haemodynamic analyses, a Millar Cather Transducer (Millar Instruments, Houston), connected to a pressure transducer (Millar Instruments), was inserted through the right carotid artery into the left ventricle cavity, and stable-state haemodynamic parameters were recorded and analyzed with LabChart software (ADInstruments, Colorado Springs, CO).

### Bone marrow derived neutrophil (BMDN) isolation and chemotaxis assay

BMDNs were isolated by Percoll gradient method as described previously (6). The purity of BMDNs was > 95% and was identified by Wright-Giemsa staining and Gr-1^+^ expression using flow cytometry, respectively.

Isolated BMDNs were re-suspended in RPMI1640 medium and pretreated with AMPK inhibitor CC (2 μM) or Nrf2 siRNA. BMDNs were then treated with LPS (5 μg/ml) for 1 h, followed with TMZ (20 μM) treatment for 2 h. After that, BMDN chemotaxis was assessed toward CXCL2 (30 ng/ml) or medium alone in a 24-well Boyden chamber using a 5-μm-pore membrane. Two hours later, the membrane was removed. The images of migrated BMDNs were captured under an optical microscope, and numbers of BMDNs were counted in at least five random fields per well.

### Immunofluorescent assay of CXCR2

Isolated BMDNs were pretreated with LPS (5 μg/ml) for 1 h, and then treated with TMZ (20 μM) for 2 h. After that, BMDNs were affixed on glass slides and incubated with anti-CXCR2 as described previously (8). BMDN nuclei were stained with DAPI. Fluorescent images were captured using fluorescence microscope (Nikon, Japan).

### Flow cytometry analysis

To determine the expression of CXCR2 on cell surface, BMDNs were stained with antibodies against FITC-CD11b, Percp/Cy5.5-Ly6G and PE-CXCR2. The expression of CXCR2 levels were analyzed by FACS Calibur flow cytometer (BD Biosciences, San Jose, CA) in the cell population of CD11b^+^Ly6G^+^.

### Transfection with siRNA

For the transfection, BMDNs were seeded in 6-well plate in optium-medium, then transfected using Lipofectamine 2,000 according to manufacturer's instruction.

### Luciferase reporter assays

Promoter fragments (−3011/+254, −2319/+254, and −1408/+254) of mouse CXCR2 were subcloned into the MluI/XholI sites of PGL3 vector (Promega, Madison, WI). The primers are shown in Supplemental Table 2. The construct of mutant CXCR2 was introduced by site-directed mutagenesis (Stratagene, La Jolla, CA). Mouse Nrf2 expression vector was purchased from Genecopoeia (EX-Mm04093-M03, Rockville, MD). All plasmids were sequenced in order to ensure sequence accuracy. Cells were transfected with CXCR2 promoter constructs and Nrf2 expressing plasmid using Lipofectamine 2000. pRL-TK (Promega, Madison, WI) was co-transfected as an internal control in each group. Forty-eight hours after transfection, cells were harvested for the Dual-Luciferase reporter assay (Promega, Madison, WI).

### Chromatin immunoprecipitation (ChIP) assays

HEK293T cells were cultured in 100-mm plates and transfected with empty or Nrf2 vector. Cells were incubated with 1% formaldehyde to cross-link protein-DNA complexes at 48 h after transfection. Cells were then lysed and sonicated to shear the chromatin to fragments. Sheared chromatin was then immunoprecipitated with anti-Nrf2 or normal IgG overnight at 4°C. Chromatin-antibody complexes were recovered by Protein A/G agroase. The immunoprecipitated DNA was analyzed by PCR to amplify CXCR2 promoter sequences. PCR products were analyzed by 1% agarose gel.

### Co-culture of BMDNS and cardiomyocytes

Primary adult cardiomyocytes were isolated as described previously (28). BMDNs seeded in 6-well plates were first administrated with SB225002 (1 μM), then stimulated with LPS (5 μg/ml) for 1 h, and finally with TMZ (20 μM) treatment for 2 h. After that, BMDNs were collected, washed, and seeded onto the transwell insert (0.4 μm pore size) above cardiomyocytes. Cardiomyocytes were harvested for 24 h at 37°C and then used for further analysis.

### Western blotting analysis

Cell lysates were generated by lysis buffer containing protease and phosphatase inhibitors. Equal amounts of protein were separated by 10% SDS-polyacyral-amide gels and transferred to PVDF membranes. Membranes were blocked with blocking buffer containing 5% BSA in TBST for 2 h at room temperature. Incubation of specific primary antibodies at 1:1000 dilutions was followed by appropriate second antibody. Then, immunoreactive bands were detected using ECL and analyzed by Quantity One software (Bio-Rad Laboratories, Philadelphia, PA).

### Statistical analysis

All animal data are presented as mean ± SEM and *in-vitro* data are presented as mean ± SD. One-way ANOVA with Bonferroni *post hoc* test was used for comparison among multiple groups using SPSS 17.0 software, and sample distribution was determined by the Shapiro–Wilk normality test (W test). Differences with *P* < 0.05 were considered statistically significant.

## Results

### TMZ protected against LPS- and CLP-induced cardiac dysfunction and promoted neutrophil migration to heart tissue

**Figure 1:**
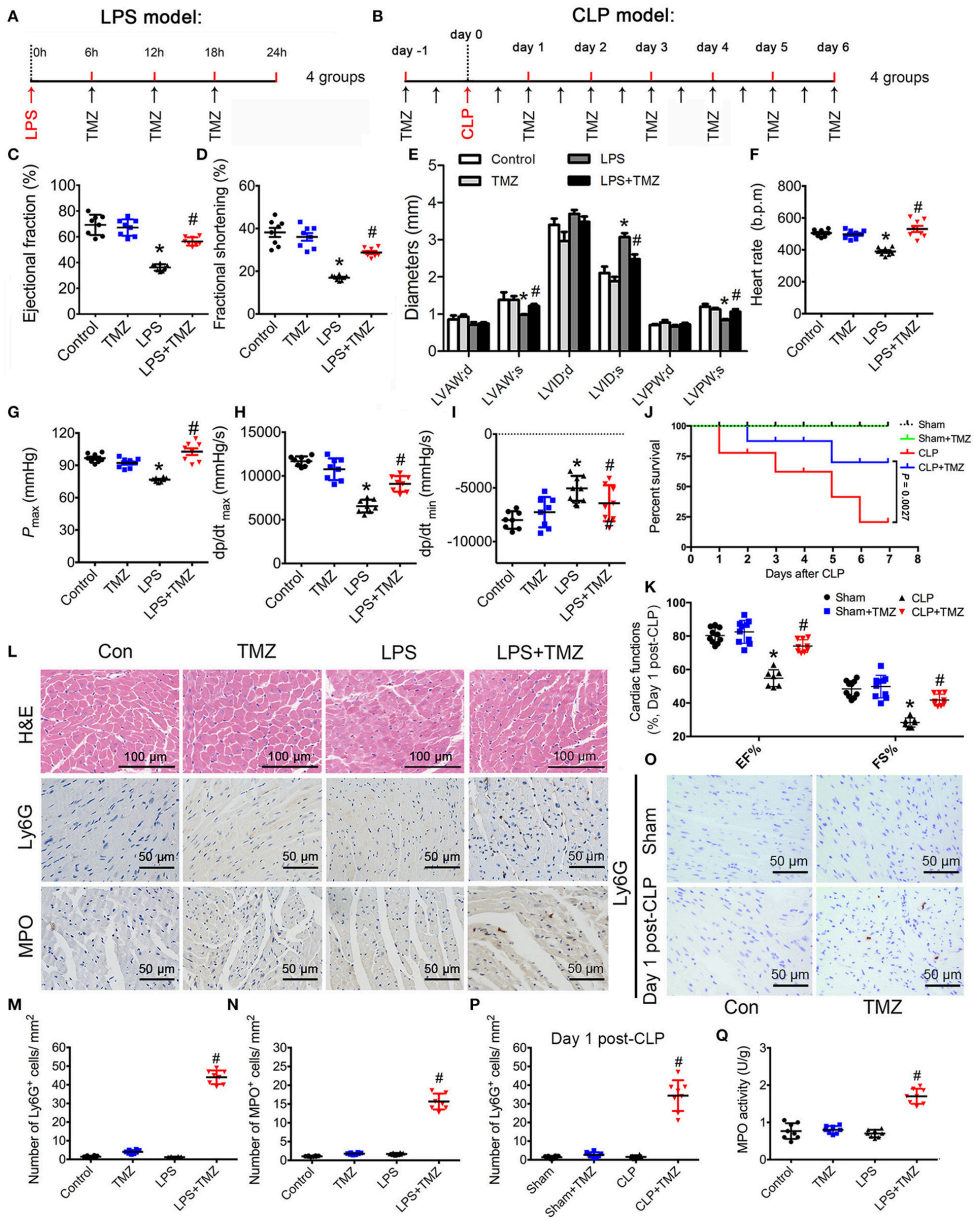
TMZ improved LPS- and CLP-induced myocardial dysfunction and increased neutrophil migration to heart tissue. **(A)** Schematic drawing of experimental schedule for the endotoxemia mice study (*n* = 8 mice per group). Mice were first injected with LPS (15 mg/kg) in 100 μl of sterile saline by i.p. Then TMZ (20 mg/kg) was administrated by i.g. every 6 h for three times at 6 h after LPS injection. 24 h after injection with LPS or saline, cardiac function of the mice was assessed by echocardiography and Millar catheter. **(B)** Schematic drawing of experimental schedule for the septic mice study (*n* = 10 mice per group). Mice were treated with TMZ (20 mg/kg/day) for 7 consecutive days post CLP or sham operations, 1 and 7 days post CLP, cardiac function of mice was assessed by echocardiography; **(C–E)** Left ventricular ejection fraction (LVEF), left ventricular fractional shortening (LVFS) and left ventricular diameters were measured by two-dimensional echocardiography. **(F–I)** Heart rate, *P*_max_, dp/dt_max_, and dp/dt_min_ were assessed by cardiac catheterization. **(J)** Survival rates of wild-type (WT) *(n* = 10 per group) undergoing severe sepsis (cecal ligation and puncture [CLP] operation). **(K)** LVEF and LVFS in CLP model were measured by two-dimensional echocardiography. **(L)** Representative histological cardiac tissues stained with H&E, Ly6G, and MPO as the indicated groups at 24 h after LPS injection. **(M)** Quantification of Ly6G positive cells in 1 mm^2^ after LPS stimulation; **(N)** Quantification of MPO positive cells in 1 mm^2^. **(O)** Representative images of heart tissues immunostained with Ly6G on day 1 post CLP. Scale bar: 50 μm. **(P)** Quantification of Ly6G positive cells in 1 mm^2^ post CLP. **(Q)** MPO activity assay in heart tissues was performed at 24 h after LPS injection. Data is presented as mean ± SEM of three independent experiments. ^*^P < 0.05 vs. Con group in LPS-induced model or Sham group in CLP-induced model; ^#^*P* < 0.05 vs. LPS-treated group or CLP-induced group. LVAW;d, left ventricular anterior wall in diastole; LVAW;s, left ventricular anterior in systole; LVID;d, left ventricular internal diameter in diastole; LVID;s, left ventricular internal diameter in diastole; *P*_max_, peak systolic pressure.

An LPS-induced endotoxemia model and a CLP-induced sepsis model were both taken in C57BL/6 mice. In the endotoxemia model, mice were injected intraperitoneally with LPS, and then either TMZ or saline (Figure 1A) was administered. In the sepsis model, mice were treated with TMZ (20 mg/kg/day) for 7 consecutive days post CLP or sham operations (Figure 1B). Consistent with our previous observations, echocardiographic parameters showed that LPS stimulation induced significant cardiac dysfunction, as indicated by decreased EF%, FS%, LVAW;s and LVPW;s, and increased LVID;s (Figures 1C–E). Similarly, haemodynamic analyses revealed that LPS injection led to decreased values of heart rate, *P*_max_, dp/dt_max_ and dp/dt_min_ (Figures 1F–I). However, TMZ administration reversed the impairments of LPS-induced cardiac functions. In addition, TMZ treatment exerted similar cardioprotective effects 1 day and 7 days post CLP surgery, including increased EF%, FS%, without affecting heart rates of mice (Figure 1K, Supplemental Figures 1A–D). Moreover, the 7-day survival rate was increased from 20.74 to 70.00% after TMZ treatment compared with CLP-induced septic mice, accompanied by reduced peritoneal bacterial load (Figure 1J and Supplemental Figure 1E), indicating the improved cardiac function after TMZ treatment confers a survival advantage in sepsis.

In histological studies, H&E staining of heart tissue in the control and TMZ group represented normal distribution of cardiomyocyte and myocardial structures (Figure 1L). Meanwhile, the LPS-induced cardiac structural disarray and interstitial edema was reversed by TMZ (Figure 1L). Ly6G and MPO staining (markers of infiltrating neutrophils) showed that the numbers of Ly6G and MPO positive cells in heart tissue was negligible in both the saline and TMZ groups, as well as in LPS- and CLP-induced mice heart. Interestingly, the number of neutrophils in heart tissue was significantly increased in the LPS+TMZ group when compared with LPS group (Figures 1L–N, Supplemental Figure 2). TMZ treatment also increased the number of neutrophils in heart tissue post CLP surgery (Figures 1N–P, Supplemental Figure 2). Meanwhile, the MPO activity in LPS+TMZ group was markedly higher than in the LPS group 24 h after LPS injection (Figure 1Q). Collectively, these results suggest that TMZ ameliorates LPS- and CLP-induced cardiac dysfunction in endotoxemia and sepsis, accompanied by increasing neutrophils recruitment into heart tissues.

### TMZ-pretreated bone marrow cells ameliorated LPS- and CLP-induced myocardial dysfunction and enhanced neutrophil recruitment to the heart

**Figure 2:**
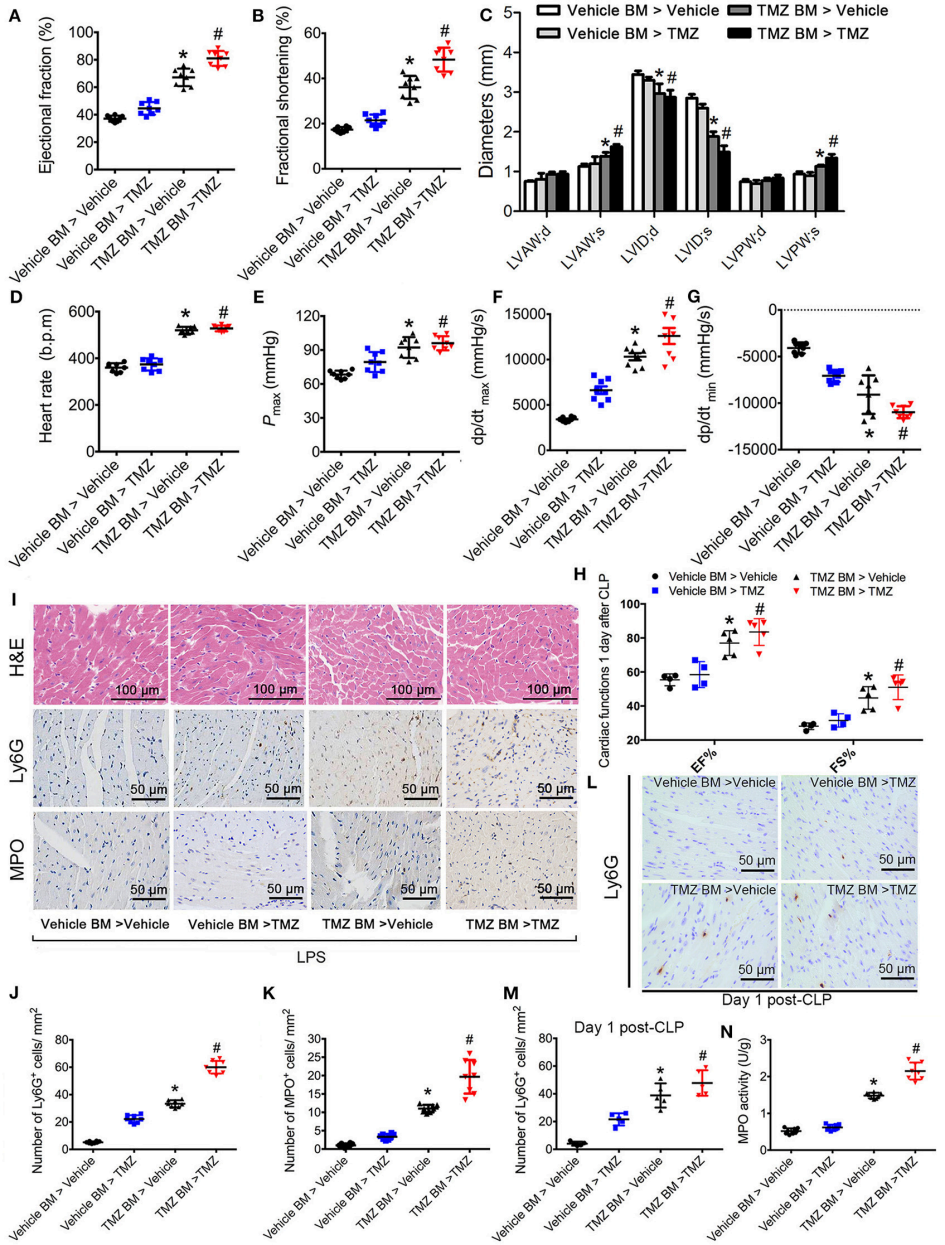
TMZ-pretreated bone marrow cells ameliorated LPS- and CLP-induced myocardial dysfunction and promoted neutrophils recruitment to heart tissues. Irradiated TMZ pretreated (20 mg/kg, i.g., Tid, for 3 days) and vehicle pretreated wild-type (WT) mice received either vehicle or TMZ pretreated bone marrow subjected to LPS challenge (15 mg/kg, i.p.) or CLP surgery, respectively. 6 h after LPS injection and 1 day post-CLP, mice were subjected to echocardiography and haemodynamic analyses. Mice were then sacrificed and heart tissues were stained with H&E and immunochemistry staining. **(A–C)** Statistical analysis of echocardiographic results after LPS challenge. **(D–G)** Statistical analysis of haemodynamic results after LPS challenge. **(H)** Statistical analysis of echocardiographic results post-CLP. **(I)** Representative histological H&E, Ly6G, and MPO staining of heart sections as the indicated groups. **(J)** Quantitative analysis of Ly6G-positive cells in 1 mm^2^ after LPS challenge. **(K)** Quantitative analysis of MPO-positive cells in 1 mm^2^ after LPS challenge. **(L)** Representative Ly6G staining of heart sections as the indicated groups in CLP-induced sepsis. **(M)** Quantitative analysis of Ly6G-positive cells in 1 mm^2^ post CLP; **(N)** MPO activity assay in heart tissues was performed at 6 h after LPS injection. Data is presented as mean ± SEM of three independent experiments. ^*^P < 0.05 vs. vehicle BM > vehicle mice; ^#^*P* < 0.05 vs. vehicle BM > TMZ mice, *n* = 8 mice per group after LPS challenge, and *n* = 5 mice per group post CLP.

To detect whether the effects of TMZ on neutrophil recruitment was dependent on resident cells or bone marrow (BM) derived cells, we performed BM transplantation experiments. Compared with mice that received vehicle BM cells, mice receiving TMZ pretreated BM cells showed improvement of cardiac function, reflected by increased values of EF%, FS%, LVAW;s, LVPW;s, heart rate, *P*_max_, dp/dt_max_ and dp/dt_min_, as well as decreased values of LVID;d and LVID;s (Figures 2A–G) post LPS stimulation. In the CLP-induced sepsis model, mice receiving TMZ pretreated BM cells showed similar improvement of cardiac function as observed in the post LPS-induced endotoxemia model (Figure 2H). Furthermore, H&E staining revealed that compared with vehicle pretreated BM cells, the TMZ pretreated BM cells that injected in recipient mice attenuated the myocardial injury, indicated by reduced irregular arrangement of cardiomyocyte and interstitial edema (Figure 2I). Moreover, in both LPS-induced endotoxemia and CLP-induced sepsis models, the Ly6G and MPO staining showed that the number of neutrophils recruited into heart tissue was significantly increased in TMZ BM > vehicle mice compared with vehicle BM > vehicle mice, and TMZ BM > TMZ mice compared with TMZ BM > vehicle mice, respectively, (Figures 2I–M). Consistently, the MPO activities in TMZ BM > vehicle and TMZ BM > TMZ groups were remarkably higher compared with vehicle BM > vehicle and TMZ BM > vehicle groups (Figure 2N). Taken together, this data indicates that TMZ-pretreated bone marrow cells attenuate LPS- and CLP-induced myocardial depression, and enhance the migration of neutrophils into heart tissue.

### TMZ enhanced neutrophil migration by regulating CXCR2 expression through AMPK pathway

**Figure 3:**
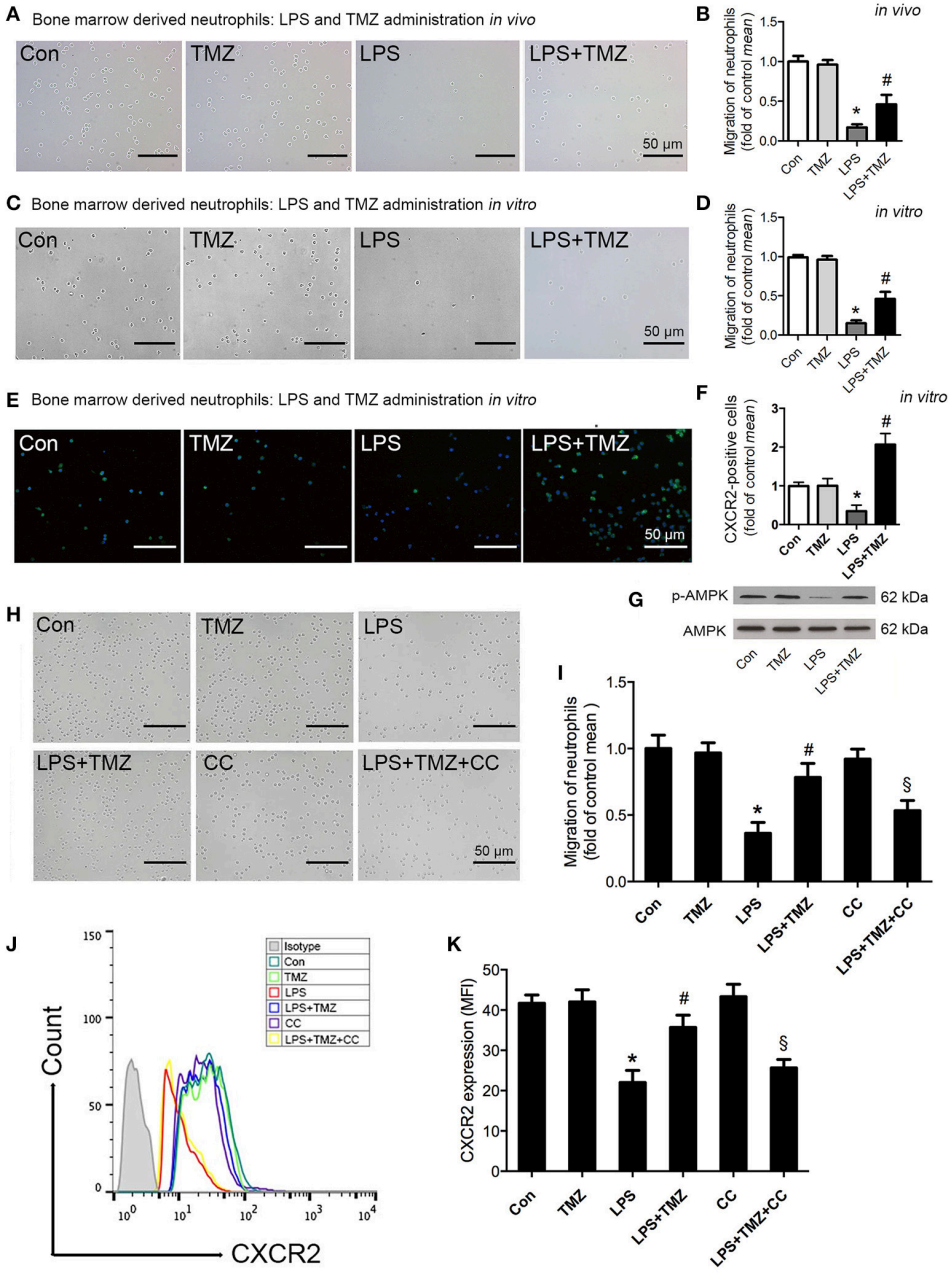
TMZ enhanced neutrophil migration by regulating CXCR2 expression through AMPK pathway. **(A)** 8–10 week-old C57BL/6 male mice were first injected with LPS (15 mg/kg), then TMZ (20 mg/kg) was administrated by gavage every 6 h for 3 times after LPS injection for 6 h. Representative images of migrated BMDNs by transwell assay. **(B)** Relative quantitative assay of migrating BMDNs under optical microscopy. **(C)** Neutrophils isolated from non-simulated mice bone marrow, then BMDNs were subjected to LPS stimulation (5 μg/ml) for 1 h and subsequently treated with TMZ (20 μM) for 2 h. Representative images of migrated BMDNs by transwell assay. **(D)** Relative quantitative assay of migrating BMDNs under optical microscopy. **(E)** Representative images of BMDN immunofluorescent CXCR2 (green) staining. Nuclei were stained by DAPI (blue). **(F)** Quantitative of CXCR2 expression by measuring fluorescence intensity. **(G)** Phosphorylation of AMPK in BMDNs was examined by western blotting. **(H)** BMDNs were first treated with AMPK inhibitor CC (CC) (1 μM) for 1 h, then subjected to LPS stimulation (5 μg/ml) for 1 h, subsequently treated with TMZ (20 μM) for 2 h *in vitro*. Representative images of migrated BMDNs by transwell assay. **(I)** Relative quantitative assay of migrating BMDNs under optical microscopy. **(J)** Flow cytometry was performed to examine the expression of CXCR2 on the membrane of neutrophils. **(K)** Quantitative of CXCR2 expression by FACS. Data were presented as mean ± SEM *in vivo* and mean ± SD *in vitro* of three independent experiments. Scale bar: 50 μm. ^*^P < 0.05 vs. Con group; ^#^*P* < 0.05 vs. LPS group; ^§^*P* < 0.05 vs. LPS+TMZ group, *n* = 8 mice per group.

To examine whether TMZ had a direct effect on neutrophil migration, we isolated bone marrow derived neutrophils (BMDNs) from differentially treated mice, and assessed the migration ability of BMDNs. As shown in Figures 3A,B, BMDNs isolated from LPS-induced mice showed a marked impaired chemotactic response toward CXCL2, when compared with the control group (29). However, TMZ treatment remarkably rescued declined BMDN migration by LPS stimulation.

Next, isolated BMDNs from C57BL/6 mice were pretreated with TMZ or LPS *in vitro*. Consistently, TMZ significantly ameliorated LPS-induced failure of BMDN migration toward CXCL2 (Figures 3C,D). Additionally, both the untreated and TMZ-treated BMDNs exhibited normal and homogeneous expression of CXCR2 in the CXCR2 fluorescent staining. However, LPS significantly reduced expression of CXCR2 in BMDNs, which was reversed by TMZ treatment (Figures 3E–F).

Next, we found that TMZ reversed LPS-reduced phosphorylation of AMPK in BMDNs (Figure 3G). Consistently, pretreatment with the AMPK specific inhibitor, CC, significantly prevented TMZ-enhanced BMDNs migration (Figures 3H–I). In addition, flow cytometry analyses showed that TMZ elevated the expression of CXCR2 on the membrane in LPS-induced BMDNs, which was reversed by CC (Figures 3J–K). Thus, the TMZ regulated CXCR2 expression in an AMPK-dependent manner, which in turn enhanced neutrophil migration.

### TMZ improved neutrophil migration by decreasing GRK2 and increasing CXCR2 expression in an AMPK/Nrf2 dependent manner

**Figure 4:**
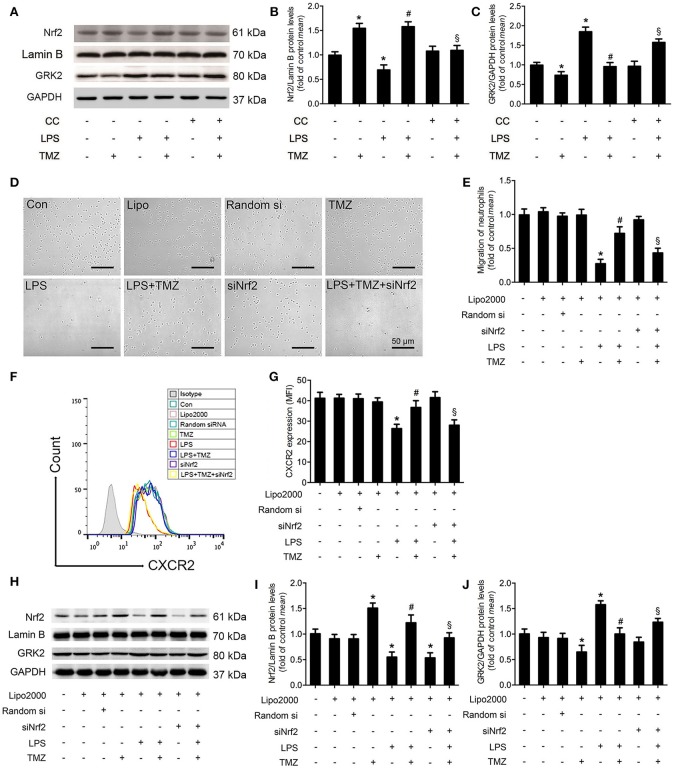
TMZ promoted neutrophil migration by decreasing GRK2 and increasing CXCR2 expression in an AMPK/Nrf2 dependent manner. **(A)** BMDNs were first treated with CC (1 μM) for 1 h, then subjected to LPS stimulation (5 μg/ml) for 1 h, subsequently treated with TMZ (20 μM) for 2 h. Western blotting analysis of nuclear Nrf2 and total GRK2 in response to different stimulations. Lamin B and GAPDH were used as loading controls, respectively. **(B,C)** Quantification of Nrf2/Lamin B and GRK2/GAPDH was performed from the western blotting and expressed as fold induction. **(D)** BMDNs were first transfected with si-Nrf2 for 24 h, then subjected to LPS stimulation (5 μg/ml) for 1 h, subsequently treated with TMZ (20 μM) for 2 h. Representative images of transwell assays for BMDNs under optical microscope. Scale bar: 50 μm. **(E)** Relative quantitative assay of migrating BMDNs under optical microscopy. **(F)** Flow cytometry was performed to examine the expression of CXCR2 on the membrane of BMDNs. **(G)** Quantitative assay of CXCR2 expression by FACS. **(H)** Western blotting analysis of nuclear Nrf2 and total GRK2 in response to different stimulations after transfection. Lamin B and GAPDH were used as loading controls, respectively. **(I,J)** Quantification of Nrf2/Lamin B and GRK2/GAPDH was performed from the western blotting and expressed as fold induction. Data is presented as mean ± SEM *in vivo* and mean ± SD *in vitro* of three independent experiments. ^*^P < 0.05 vs. Con group; ^#^*P* < 0.05 vs. LPS group; ^§^*P* < 0.05 vs. LPS+TMZ group.

As shown in Figures 4A,B, we found that TMZ markedly reversed the reduced Nrf2 expression in the nucleus of LPS-treated BMDNs, whereas CC abrogated this influence of TMZ. On the other hand, by Western blotting analyses, we found that TMZ treatment significantly inhibited LPS-induced GRK2 over-expression, which was attenuated by CC, indicating that TMZ negatively affected GRK2 expression via AMPK activation (Figures 4A,C). To verify whether Nrf2 regulates BMDN migration, BMDNs were transfected with Nrf2 siRNA. In LPS-treated BMDNs, Nrf2 silence significantly prevented the effects of TMZ on enhancing neutrophil migration (Figures 4D,E), downregulating CXCR2 membrane expression (Figures 4F,G), inhibiting Nrf2 expression (Figures 4H,I), and increasing GRK2 expression (Figures 4H,J).

### CXCR2 is transcriptionally regulated by TMZ via Nrf2 in LPS-induced cardiac dysfunction

**Figure 5:**
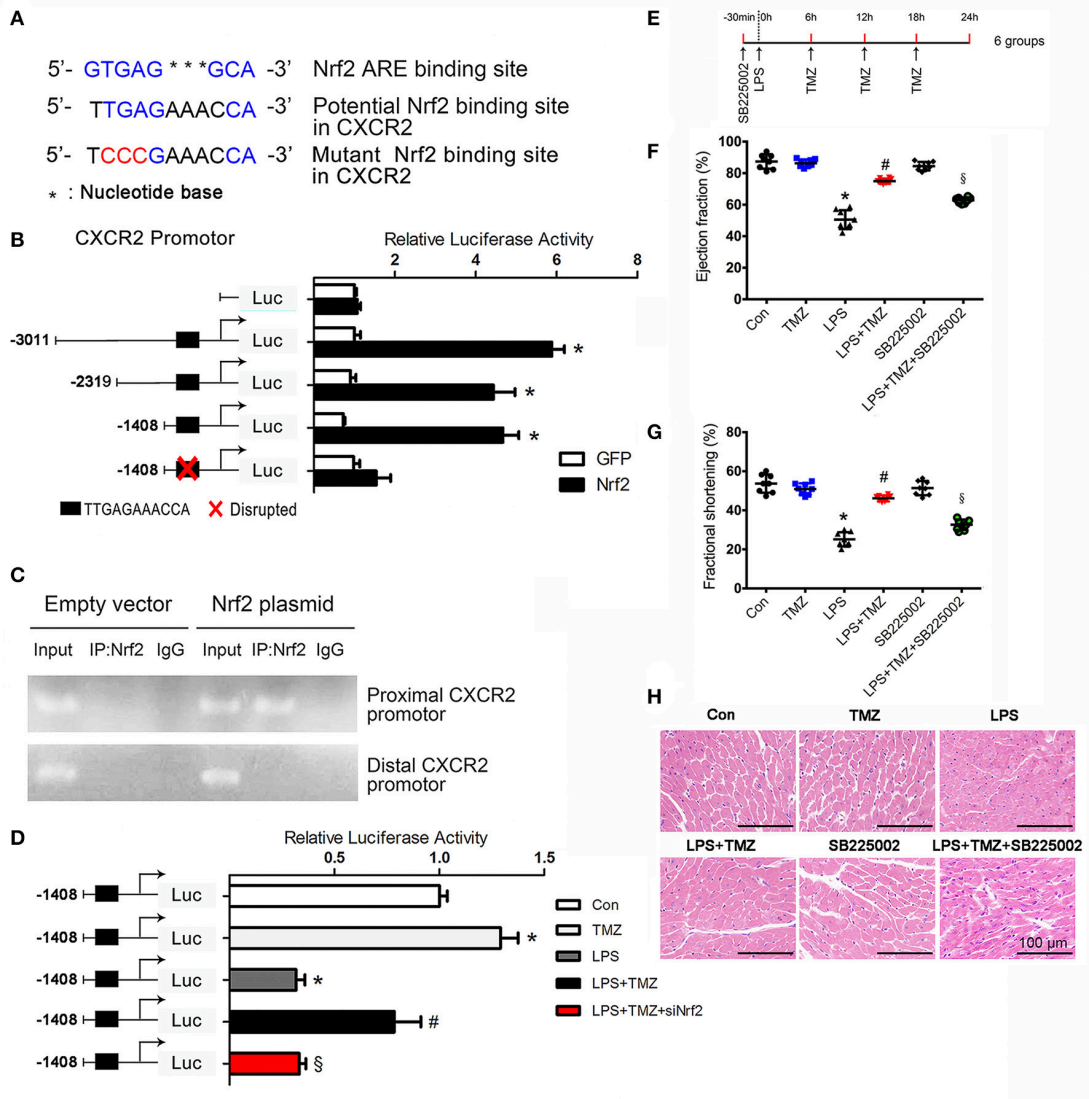
CXCR2 is transcriptionally regulated by TMZ via Nrf2 in LPS-induced cardiac dysfunction. **(A)** Nrf2 ARE consensus binding site, potential Nrf2 binding site and the mutant Nrf2 binding site relative to potential Nrf2 binding site on mouse CXCR2. Blue indicates binding site, red indicates mutated base. **(B)** Luciferase activity of the constructs transfected into HEK293T cells. The first base before ATG represents−1. **(C)** Nrf2 chromatin immunoprecipitation. HEK293T cells were transfected with an empty plasmid or plasmid expressing Nrf2. PCR assays on input and IP fractions amplified the CXCR2 promoter containing the putative Nrf2 site (−919/−909, top panel) or a distal region of the Nrf2 promoter (−2727/−2717, bottom panel). **(D)**−1408/+254 CXCR2 promoter constructs and si-Nrf2 were transfected into HEK293T cells, and then subjected to TMZ (20 μM), LPS (5 μg/ml) or combination stimulations. Relative luciferase activity of−1408/+254 CXCR2 promoter construct after stimulations. **(E)** Schematic drawing of experimental schedule for the mice study. 8–10 week-old C57BL/6 male mice were first intraperitoneally injected with the CXCR2 inhibitor SB225002 (10 mg/kg) and LPS (15 mg/kg), then TMZ was administrated by gavage every 6 h for 3 times after LPS injection for 6 h. **(F,G)** Ejection fraction and fractional shortening was measured by two-dimensional echocardiography. **(H)** Representative images of left ventricular myocardium H&E staining. Scale bars: 100 μm. Data is presented as mean ± SEM *in vivo* and mean ± SD *in vitro* of three independent experiments. ^*^P < 0.05 vs. corresponding Control group or GFP control; ^#^*P* < 0.05 vs. LPS group; ^§^*P* < 0.05 vs. LPS+TMZ group. *n* = 8 mice per group.

To understand how Nrf2 affects the expression of CXCR2, we performed *in silico* promoter analyses on mouse CXCR2 gene. Bioinformatic analyses revealed a CXCR2 potential ARE (antioxidant responsive element) binding sequence upstream of the CXCR2 translational initiation site (−919/−909). Subsequently, three fragments (−3011/+254, −2319/+254, −1408/+254) of promoter sequence of CXCR2 and an ARE mutant fragment of −1408/+254 were inserted into the luciferase reporter plasmid (Figures 5A,B). Luciferase reporter gene assays revealed that Nrf2 increased the luciferase activity of all three fragments, but not in the mutant fragment (Figure 5B), indicating that Nrf2 affected the transcriptional activity of CXCR2 via binding to the potential ARE sequence. To confirm this binding, we performed chromatin immunoprecipitation assays. In agreement with the results of luciferase reporter gene assays, the CXCR2 proximal promoter (−919/−909) was present in Nrf2 immunoprecipitates from cells expressing Nrf2, but not cells expressing the empty vector control. In contrast, distal promoter sequences (−2727/−2717) could not be amplified in Nrf2 immunoprecipitates (Figure 5C). Moreover, TMZ increased transcriptional activity of CXCR2 in the proximal promoter sequence from −1408 to +254 compared with LPS, and this effect was dependent on the expression of Nrf2 (Figure 5D). Together, this data demonstrates that CXCR2 is transcriptionally regulated by TMZ through the transcription factor Nrf2.

To test whether the expression of CXCR2 in neutrophils contributes to the protective effects of TMZ against LPS-induced cardiac dysfunction, a specific CXCR2 antagonist, SB225002, was introduced to inhibit the expression of CXCR2 *in vivo* (Figure 5E). Results showed that TMZ significantly ameliorated LPS-induced cardiac dysfunction, whereas CXCR2 antagonist almost completely abrogated the protective effects of TMZ in LPS-induced mice (Figures 5F–H and Supplemental Table 1). These results indicate that TMZ prevents LPS-induced cardiac depression via up-regulating the expression of neutrophil's CXCR2.

### TMZ decreased LPS-induced cardiomyocyte pyroptosis by targeting neutrophils

**Figure 6:**
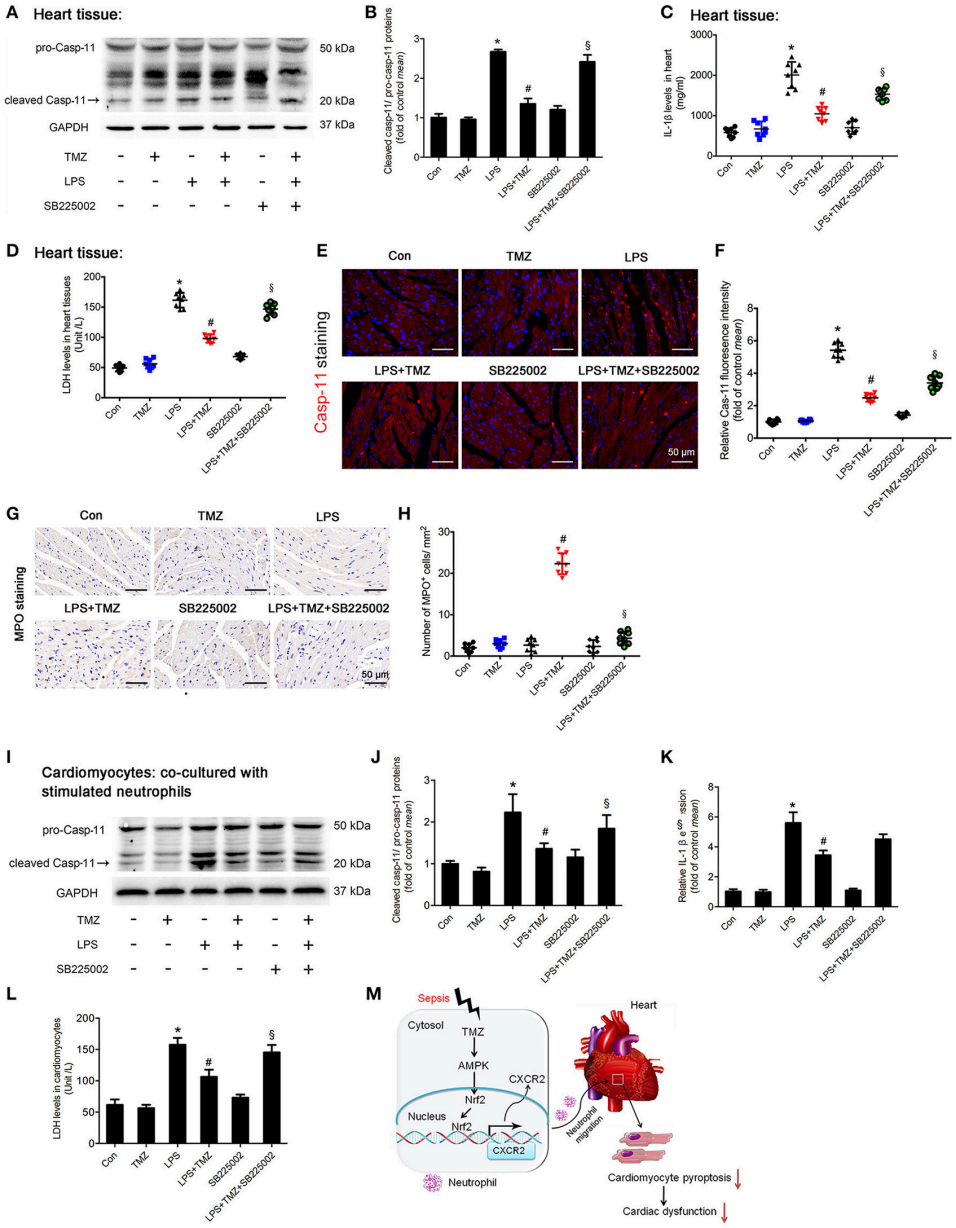
TMZ decreased LPS-induced cardiomyocyte pyroptosis by targeting neutrophils**. (A)** 8–10 week-old C57BL/6 male mice were first injected with the CXCR2 inhibitor SB225002 (10 mg/kg) and LPS (15 mg/kg), then TMZ was administrated by gavage every 6 h for 3 times after LPS injection for 6 h. Cleaved caspase-11 (marked by arrow) expression in heart tissue was measured by western blotting. **(B)** Quantification of cleaved Casp-11/ pro-Casp-11 was performed from the western blotting analysis and expressed as fold induction. **(C)** IL-1β levels in heart tissues were measured by ELISA. **(D)** LDH levels in heart tissue. **(E)** Representative images of left ventricular myocardium caspase-11 (red) fluorescent staining. Blue indicates DAPI staining. Scale bar: 50 μm. **(F)** Relative caspase-11 fluorescent intensity. **(G)** Representative images of left ventricular myocardium MPO staining. Scale bar: 50 μm. **(H)** Quantification of MPO-positive cells in 1 mm^2^. **(I)** BMDNs were first administrated with SB225002 (1 μM), then stimulated with LPS (5 μg/ml) for 1 h, and finally with TMZ (20 μM) treatment. Primary cardiomyocytes of adult mice were co-cultured with stimulated BMDNs by transwell. Cardiomyocytes were seeded in the bottom chamber and BMDNs into the upper chamber. Western blotting analysis of Casp-11 in cardiomyocyte and GAPDH used as loading control. **(J)** Quantification of cleaved Casp-11/ pro-Casp-11 was performed from the western blotting analysis and expressed as fold induction. **(K)** Relative expression of IL-1β mRNA in cardiomyocyte (normalized to GAPDH mRNA). **(L)** LDH levels in cardiomyocytes. **(M)** The proposed model for this study was summarized. Data is presented as mean ± SEM *in vivo* and mean ± SD *in vitro* of three independent experiments. ^*^P < 0.05 vs. corresponding Control group; ^#^*P* < 0.05 vs. LPS group; ^§^*P* < 0.05 vs. LPS+TMZ group.

Inflammatory response in some pathological conditions can induce cardiac cell death, subsequently leading to heart failure, which is associated with pyroptosis (13). Activation of caspase-11 (cleaved caspase-11) and marked increase of IL-1β and LDH were served as biomarkers of pyroptosis (22, 30). In this study, Western blotting showed that the expression of cardiac cleaved caspase-11 was remarkably increased in the LPS group (Figures 6A,B), and similar changes in IL-1β and LDH were consistently observed (Figures 6C,D). Increased caspase-11 in the LPS stimulated heart was also detectable by fluorescence staining (Figures 6E,F). These results indicated an induction of cardiomyocyte pyroptosis in LPS stimulated septic mice. As expected, TMZ treatment effectively reduced LPS-induced cardiomyocyte pyroptosis (Figures 6A–F). On the other hand, the specific CXCR2 antagonist, SB225002, attenuated the protective effects of TMZ on LPS-induced cardiomyocyte pyroptosis (Figures 6A–F). Moreover, the protective effects of TMZ on LPS-induced cardiac pyroptosis were associated with increased neutrophils in heart tissue (Figures 6G,H). Consistently, in *in-vitro* experiments, cardiomyocytes co-cultured with LPS-stimulated neutrophils exhibited significant pyroptosis, reflected by increased levels of cleaved caspase-11, IL-1β, and LDH (Figures 6I–L). Conversely, TMZ-pretreated BMDNs decreased cardiomyocyte pyroptosis induced by LPS, indicating a protective effect of TMZ on cardiomyocyte pyroptosis mediated by neutrophils (Figures 6I–L). After adding the CXCR2 specific inhibitor SB225002, the protective effects of TMZ on LPS-induced cardiomyocyte pyroptosis were blocked (Figures 6I–L). This data suggests that TMZ attenuates LPS-induced cardiomyocyte pyroptosis via neutrophils mediated by CXCR2 *in vivo* and *in vitro*.

## Discussion

In the current study, we found that the anti-anginal agent TMZ significantly attenuated LPS- and CLP-induced myocardial dysfunction in mice, by increasing neutrophilic migration to heart tissue (Figure 6M), via promoting neutrophil recruitment to the heart. Mechanistically, we found that TMZ promoted neutrophils through an AMPK/Nrf2/CXCR2 dependent manner secondary to LPS stimulation. CXCR2 was transcriptionally regulated by TMZ via Nrf2, which directly bound to the CXCR2 promoter sequence. Finally, TMZ reduced LPS-induced cardiomyocyte pyroptosis via neutrophils.

Sepsis and endotoxemia are both systemic inflammatory responses to infection and can lead to tissue injury and multiple organ failure (31–33). The cardiovascular system is one of the most frequently affected organ systems in sepsis and endotoxemia (33, 34). Septic patients with secondary cardiac dysfunction had a 50-70% increase in mortality when compared to those without cardiac dysfunction (3). The myocardial contractile dysfunction is driven by several factors, such as cardiodepressant mediators, mitochondrial dysfunction and/or apoptosis (35, 36). During sepsis-induced cardiac dysfunction, various cell types and factors involved in the up-regulation of inflammatory gene transcription and initiation of innate immunity in heart (3). Our previous study demonstrated the protective role of TMZ against LPS-induced cardiac dysfunction, by targeting the macrophage mediated pro-inflammatory response, especially through activating macrophages in bone marrow (14). In this study, we found that the myocardial beneficial effects of TMZ were associated with increased neutrophil recruitment after LPS challenge. Furthermore, in bone marrow transplantation experiments, we identified that TMZ pre-treated bone marrow cells, other than resident cells, prevented LPS-induced cardiac dysfunction and promoted neutrophil recruitment into the heart. Even though TMZ was pre-treated in the group vehicle BM > TMZ, vehicle pre-treated bone marrow cells did not prevent LPS- or CLP-induced cardiac dysfunction and failed to promote neutrophil recruitment into the heart. Given that CXCR2 plays an important role in the retention and release of neutrophils in bone marrow (37), we speculate that the role of TMZ is different in bone marrow versus peripheral tissue (heart), and TMZ mainly acts in bone marrow.

To elucidate the functions of increased neutrophils in heart tissue, we assessed the ability of neutrophil migration. Migration of neutrophils was regulated by corresponding chemokines via binding to the specific chemokine receptors, which belong to the G protein-coupled receptor family (38). CXCR2 plays an important role in mice neutrophil recruitment into the infected site (39). The failure of neutrophil migration was associated with CXCR2 depression (8). GRK2 is a serine/threonine proteins kinase and its activation increased the internalization of the chemokine receptor CXCR2 (9). However, the detailed mechanism underlying the direct regulation of CXCR2 expression on the membrane of neutrophils during sepsis has been poorly investigated. Previous studies have reported that AMPK activation enhances neutrophil chemotaxis and bacterial killing (12). The present study demonstrates that TMZ promoted LPS-inhibited BMDN migration *in vitro* is accompanied by an increase in CXCR2 membrane expression, which was significantly prevented by the AMPK inhibitor CC. Moreover, TMZ induced accumulation of Nrf2 in the nucleus in BMDNs contributed to the increased CXCR2 membrane expression. Additionally, the suppression of GRK2 induced by TMZ also reduced the rapid internalization of CXCR2.

When cells were exposed to oxidative or electrophilic stress, Nrf2 disassociated from Keap1 and translocated into the nucleus, where it was able to regulate the transcription of its target genes by binding to the anti-oxidative response element (ARE) located in the promoter area (13). Nrf2 regulates antioxidant genes, resulting in elimination of reactive oxygen species (ROS) and decreased inflammation (40). Recently, Nrf2 has been evidenced to bind to the pro-inflammatory genes IL-6 and IL-1, blocking their transcriptions in macrophages (41). Moreover, Nrf2 activation protected cardiac tissue from injury caused by diabetic cardiomyopathy (42). In this study, for the first time, we provided evidences that Nrf2 directly binds to a new site located in the CXCR2 promoter sequence, resulting in higher expression of CXCR2 on the membrane and increased neutrophil migration. Furthermore, TMZ significantly enhanced the LPS-inhibited transcriptional activity of CXCR2, while blockage of Nrf2 abrogated the effects of TMZ. In addition, blockage of CXCR2 *in vivo* inhibited the cardiac protective effects of TMZ in LPS-stimulated mice.

Sepsis is the systemic inflammatory response to infection and the inflammatory process contributed to the occurrence of pyroptosis (1). Whether or not cardiomyocyte pyroptosis is involved in sepsis-induced cardiac dysfunction still needs to be elucidated. Pyroptosis is a highly pro-inflammatory form of programmed cell death that occurs in response to diverse organism insults. Pyroptosis occurs when canonical inflammasomes, including NLRP1, NLRP3, NLRC4, and AIM2 activates caspase- 1. On the other hand, activation of noncanonical inflammasomes is triggered by intracellular LPS directly binding to the murine caspase-11 and its human counterparts, caspase-4 and caspase-5 (22). Inhibition of caspase-1 protected against inflammation and cardiac dysfunction that results from myocardial infarction (MI) (43). Recent studies have revealed that attenuation of cardiomyocyte pyroptosis effectively ameliorates diabetic cardiomyopathy (13). Our results showed the involvement of pyroptosis in the heart of LPS-treated mice, indicated by activation of caspase-11, IL-1β release, and LDH release. Interestingly, TMZ significantly suppressed LPS-induced pyroptosis, which was reversed by the CXCR2 antagonist, SB225002. Combined with MPO staining, these results suggest that increased neutrophil recruitment is associated with decreased cardiomyocyte pyroptosis. The direct recruitment of neutrophils to the injured tissue is essential to eliminate the pathogen. However, in some specific condition (just like pyroptosis, a form of inflammatory programmed cell death), the neutrophils were pronounced accumulated in heart to help opsonization of pore-induced intracellular traps (PITs). The PIT initiated a robust and coordinated innate immune response involving multiple mediators that attracted neutrophils to efferocytose the PIT (44, 45). The enhanced neutrophils recruitment would effectively contribute to the attenuation of pyroptosis via efferocytosing the PIT. TMZ may reduce pyroptosis via efferocytosing the PIT, which is mediated by the enhanced neutrophils. Considering the complex inflammatory environment during lytic cell death, we reasoned that additional signals likely contributed to neutrophil recruitment. Indeed, besides inducing pyroptosis, caspase-1 also induces secretion of IL-1β and IL-18. IL1β^−/−^IL18^−/−^ mice had significantly reduced neutrophil recruitment after the induction of pyroptosis in the tissue (44, 46). Thus, the normal caspase-1 induced pyroptosis led to secretion of IL-1β and IL-18, which could increase neutrophil recruitment. Besides inflammatory cytokines, the CXCL2 and CXCR2 signaling pathway also contributed to the neutrophil recruitment. In our current study, we mainly focused on the CXCR2 signaling pathway and investigated the role of TMZ during sepsis. We speculate that neutrophil mediated efferocytose of the PITs would play a corresponding role during LPS-induced cardiomyocyte pyroptosis.

In conclusion, our results showed that TMZ attenuated LPS-induced cardiomyocyte pyroptosis and cardiac dysfunction by promoting neutrophil recruitment to cardiac tissue via CXCR2. This suggests that TMZ may be a potential drug for the treatment of septic cardiac dysfunction.

## Author contributions

CC and DWW: conception and design. JC and BW: experiments, analysis, and draft writing. JL, MH, GR, ZY, and JW: part experiments. ZB, KC, and QN: English editing.

### Conflict of interest statement

The authors declare that the research was conducted in the absence of any commercial or financial relationships that could be construed as a potential conflict of interest.

## Abbreviations

NADPH: nicotinamide adenine dinucleotide phosphate
EF: ejection fraction
FS: fraction shortening
LVAW: left ventricle anterior wall
LVPW: left ventricle posterior wall
dP/dt max: peak instantaneous rate of left ventricular pressure increase
dP/dt min: peak instantaneous rate of left ventricular pressure decline
LPS: lipopolysaccharide
MPO, myeloperoxidase. TMZ: trimetazidine.
