# Supplementary material for: Trimetazidine Attenuates Cardiac Dysfunction in Endotoxemia and Sepsis by Promoting Neutrophil Migration
Source: Front Immunol. 2018 Sep 4;9:2015. doi: 10.3389/fimmu.2018.02015 (PMC6131494; doi:10.3389/fimmu.2018.02015)
Supplement: Supplementary file 1 [file Data_Sheet_1.pdf]

Supplementary materials

Trimetazidine attenuates cardiac dysfunction in endotoxemia and sepsis by promoting neutrophil migration

Jing Chen#, M.D., Ph.D.; Bei Wang#, M.D., Ph.D.; Jinsheng Lai, M.D., Ph.D.; Zachary Braunstein, MD.; Mengying He, M.D.; Guoran Ruan, M.D.; Zhongwei Yin, M.D., Ph.D.; Jin Wang, M.D.; Katherine Cianflone, Ph.D.; Qin Ning, M.D., Ph.D., Chen Chen, M.D., Ph.D. and Dao Wen Wang, M.D., Ph.D.

Supplementary Figure 1:

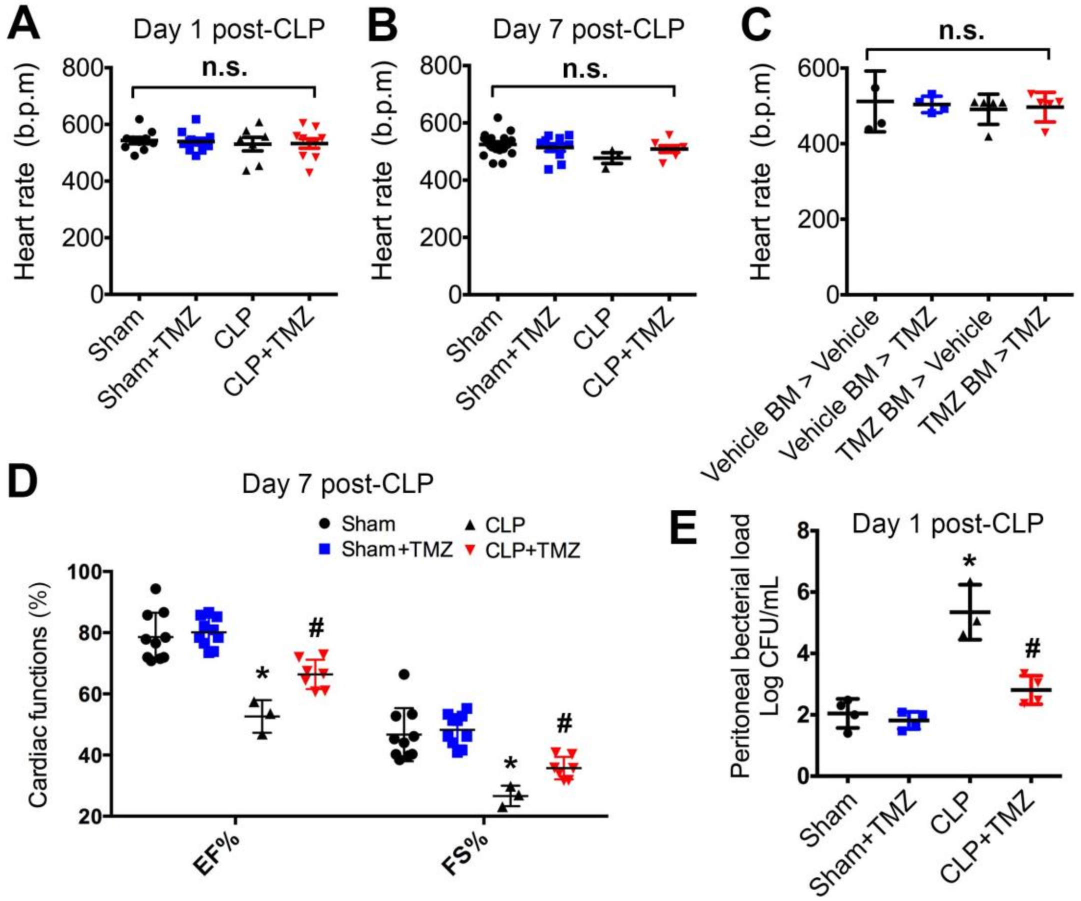

Supplementary Figure 1: TMZ treatment attenuates cardiac function post-CLP. Mice were treated with TMZ (20 mg/kg/day) for 7 consecutive days post CLP or sham operation. 1 day and 7 days post CLP, the cardiac function of mice was assessed by echocardiography; (A) Heart rate on day 1 post CLP; (B) Heart rate on day 7 post CLP; (C) Irradiated TMZ pretreated (20 mg/kg/day) and vehicle pretreated wild-type (WT) mice received either vehicle or TMZ pretreated bone marrow and were subjected to CLP surgery. Heart rate on day 1 post-CLP. (D) Statistical analysis of echocardiographic results post-CLP. (E) Colony-forming units (CFU) in peritoneal exudates 1 day after CLP. Data were presented as mean ± SEM of three independent experiments. *P < 0.05 vs. Sham mice; #P < 0.05 vs. CLP mice. The n.s. indicates no significance.

Supplementary Figure 2:

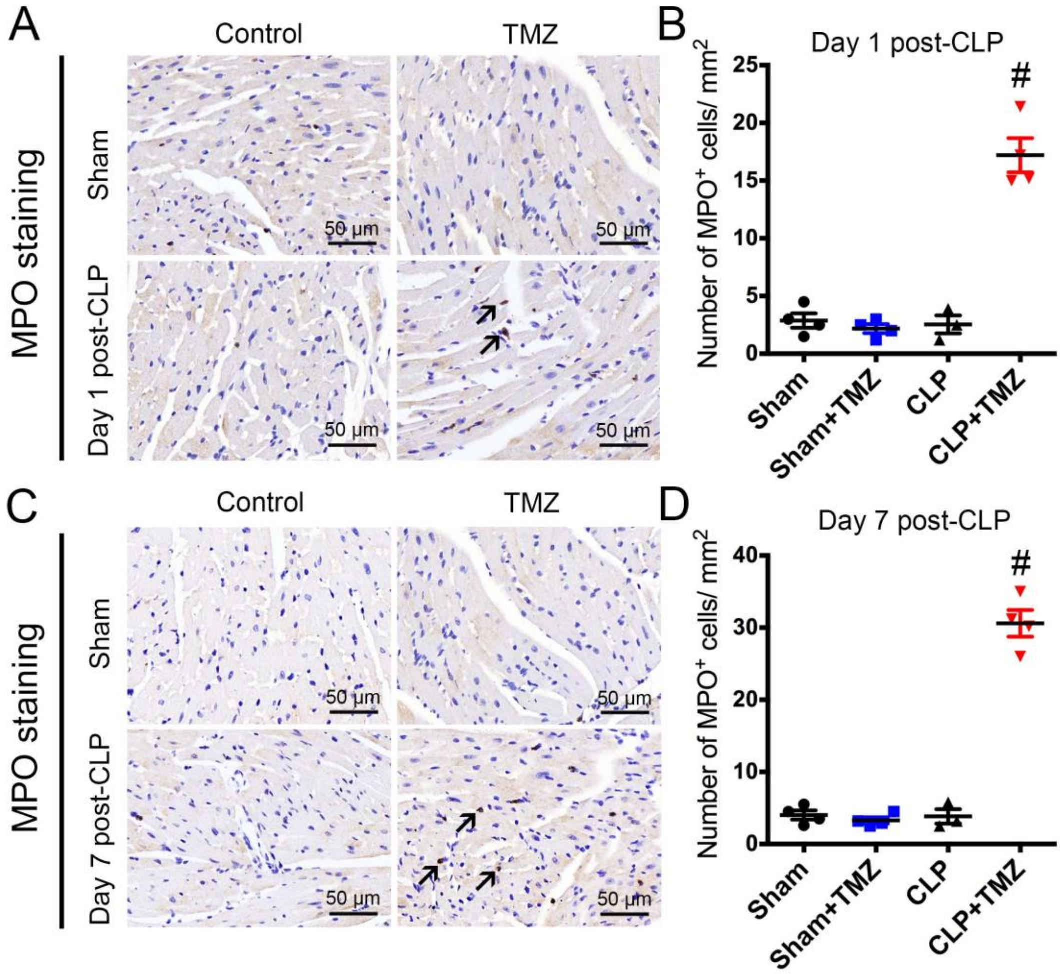

Supplementary Figure 2: TMZ increased neutrophil migration to heart tissue in CLP-induced sepsis. Mice were treated with TMZ (20 mg/kg/day) for 7 consecutive days post CLP or sham operation, 1 day and 7 days post CLP. (A) Representative images of heart tissues immunostained with MPO on day 1 post-CLP. Scale bar: 50 μm. (B) Quantification of MPO positive cells in $1mm^2$ on day 1 post-CLP. (C) Representative images of heart tissues immunostained with MPO on day 7 post-CLP. Scale bar: 50 μm. (D) Quantification of MPO positive cells in $1mm^2$ on day 7 post-CLP. Data were presented as mean ± SEM of three independent experiments. #P < 0.05 vs. CLP mice. (n = 3-4 per each group)
