# Supplementary material for: Trimetazidine Attenuates Cardiac Dysfunction in Endotoxemia and Sepsis by Promoting Neutrophil Migration
Source: Front Immunol. 2018 Sep 4;9:2015. doi: 10.3389/fimmu.2018.02015 (PMC6131494; doi:10.3389/fimmu.2018.02015)
Supplement: Supplementary file 2 [file Data_Sheet_2.pdf]

Supplemental Table 1. Haemodynamic characteristics of animal groups.

	Con	TMZ	LPS	LPS+TMZ	SB225002	LPS+TMZ+SB225002
Heart rate (b.p.m.)	501.1 ± 7.39	494.3 ± 9.93	330.5 ± 12.27*	459.3 ± 7.69$^{\#}$	506.4 ± 3.88	364.3 ± 8.07§
P_{max} (mmHg)	106.8 ± 3.28	107.9 ± 3.52	66.6 ± 2.67*	94.5 ± 1.86$^{\#}$	108.8 ± 4.38	77.69 ± 1.31§
EDP (mmHg)	4.64 ± 0.31	4.63 ± 0.22	14.26 ± 1.32*	5.76 ± 0.30$^{\#}$	4.70 ± 0.29	8.38 ± 0.75§
dP/dt_{max} (mmHg/s)	11747 ± 381.7	11799 ± 918.8	4949 ± 258.2*	8628 ± 209.3$^{\#}$	11475 ± 897.9	5726 ± 324.6§
dP/dt_{min} (mmHg/s)	−6034 ± 142.4	−5813 ± 365.9	−3413 ± 260.3*	−5534 ± 188.2$^{\#}$	−5737 ± 421.1	−3713 ± 237§

Values represent mean ± SEM; n =7-8 per group.

HR, heart rate; Pmax, peak systolic pressure; EDP, end diastolic pressure; dP/dt max, peak instantaneous rate of left ventricular pressure increase; dP/dt min, peak instantaneous rate of left ventricular pressure decline.

$^{*}p$ <0.05 Con group, $^{\#}p$ <0.05 LPS group, $^{§}p$ <0.05 LPS+TMZ group.

Supplemental Table 2. Primers for CXCR2 promoter constructs.

Constructs	Sequences	Forward or 5' primer	Reverse or 3' primer
CXCR2 promoter construct1	(-3010) ~ +252	CTGGGGATGTGATGGGAGAG	TAGACATCGGTGACAGAGCAG
CXCR2 promoter construct2	(-2318) ~ +252	GGGGCATTTATGGGCTGTAT	TAGACATCGGTGACAGAGCAG
CXCR2 promoter construct3	(-1407) ~ +252	CTTTTATCCAACCAGCCACTT	TAGACATCGGTGACAGAGCAG
